# Reply to: “The partner-an underutilized facilitator to support healthy gestational weight gain”

**DOI:** 10.1186/s12884-023-05713-3

**Published:** 2023-06-15

**Authors:** Tamara Escañuela Sánchez, Sarah Meaney, Keelin O’Donoghue, Molly Byrne, Karen Matvienko-Sikar

**Affiliations:** 1 Pregnancy Loss Research Group, Department of Obstetric and Gynaecology, University College Cork, Cork, Ireland; 2 National Perinatal Epidemiology Centre (NPEC), Deparment of Obstetric and Gynaecology, University College Cork, Cork, Ireland; 3 INFANT Centre, University College Cork, Cork, Ireland; 4 Health Behaviour Change Research Group, School of Psychology, NUI Galway, Galway, Ireland; 5 School of Public Health, University College Cork, Cork, Ireland

## Abstract

Facilitators and barriers influencing weight management behaviours were identified in our meta-synthesis of qualitative research entitled “Facilitators and barriers influencing weight management behaviours during pregnancy: a meta-synthesis of qualitative research”. This manuscript is in response to the letter submitted by Sparks et al. regarding that work. The authors highlight the importance of including partners into intervention design when addressing weight management behaviours. We agree with the authors that it is important to include partners into intervention design and further research is granted to identify facilitators and barriers affecting their influence over women. As per our findings, the influence of the social context goes beyond the partner and we suggest that future interventions should address other relevant people in women’s contexts such as parents, other relatives, and close friends.

## Body of work

We would like to thank the authors for their careful reading of our qualitative evidence synthesis and for their thoughtful letter and consideration [1].

The authors correctly comment that most interventions designed to date to address weight management during pregnancy are focused on the woman only and the authors also highlight the importance of including other influences over the woman’s behaviour. These influences can be identified in different layers in society as described by the Social Determinants of Health Model and in our paper entitled “Facilitators and barriers influencing weight management behaviours during pregnancy: a meta-synthesis of qualitative research” [2]. For example, we identified barriers associated with the individual women such as lack of skills to cook healthy meals and or negative or defensive attitudes towards advice, issues related to economic stability (affordability of exercise facilities or healthy food), safety and transportation (neighbourhood unsafety that prevented women from exercising outdoors), access to healthy options (lack of healthy food stores within walking distance), education (lack of health literacy) and with the healthcare system (feelings of rejection and stigmatisation in healthcare system, education opportunities missed). This was in addition to factors associated with women’s social context, from the influence of their closer family members to a wider societal influence affecting their behaviours through social stigmatisation.

In the first instance, we agree that the influence of the social context is crucial in women’s process of behaviour change, as stated in our paper and in other studies from our group, including partners and other relevant people [3, 4]. The authors mention some findings from our study that highlight the need to conduct further research into addressing partners’ facilitators and barriers to support women’s behaviour change, and the need to include partners in future intervention design. We would like to directly respond to the authors’ comment that one of our findings shows that “most women had no decision-making power over shopping or cooking choices in the family”. We would like to clarify that this finding was supported by three studies included in our review and mostly referred to women living in multi-generational households. This nuance is illustrated in the quote supporting this finding: “*The food that my mother buy [gets in the way of me reaching my GWG goal]. She don’t buy healthy food, she, cuz my brother, and they all like fried chicken… She don’t buy plain chicken*” [5]. As such, this finding is specific to a sub-group of women included in the reviewed studies and is not intended to represent all women’s experience. We agree that many women act as “nutrition gatekeepers” in their households as stated previously in the literature [6, 7], however, in scenarios where the pregnant woman might not adopt that role because of others with stronger authority in their households, it can act as a barrier influencing their weight management behaviours, as stated in our review.

We agree with the authors that engaging partners in prenatal interventions represents a good opportunity to encourage and achieve optimal nutrition during pregnancy and physical activity goals. In addition to this, our findings show that the influence of the social context goes beyond the partner, and future interventions should also ideally address other relevant people like parents, other relatives and close friends.

In conclusion, we agree with the authors on the importance of including the closer social context of the woman in future intervention design, including partners, parents, or any of the different direct social influences over the women. One of the gaps that previous research has identified as potentially hindering the effectiveness of weight management interventions during pregnancy is the lack of involvement of the wider family and social networks surrounding the pregnant woman, in order to increase education [8]. Hence, as the authors state in their letter, it is important to conduct further research to gain insight into the facilitators and barriers that these different social influences observe when trying to support pregnant women to engage in weight management behaviours. Similar to the work conducted by Keely et al [9], which involves partners views and social networks into their exploration of the attitudes and health behaviours during pregnancy of women with a high BMI [9], this can ensure that all relevant people’s needs are addressed when supporting women’s prenatal weight management behaviours.

## Data Availability

Not applicable.
